# P-327. Geospatial and temporal trends in HIV pre-exposure prophylaxis use across metropolitan counties of the United States from 2016 through 2022

**DOI:** 10.1093/ofid/ofaf695.546

**Published:** 2026-01-11

**Authors:** Ronit Gupta, Johnny Yue, Ribhav Gupta

**Affiliations:** Harvard University, Boston, Massachusetts; Yale University, New Haven, Connecticut; Stanford University, Stanford, CA

## Abstract

**Background:**

HIV Pre-exposure prophylaxis (PrEP) remains a highly effective tool in HIV prevention. While studies on national gains in PrEP access, little is known about how access has over time and geography sub-nationally. We characterize trends in PrEP access across U.S. metropolitan counties from 2016-2022, identifies regional disparities, and evaluate the Ending the HIV Epidemic (EHE) initiative.

**Figure 1.**
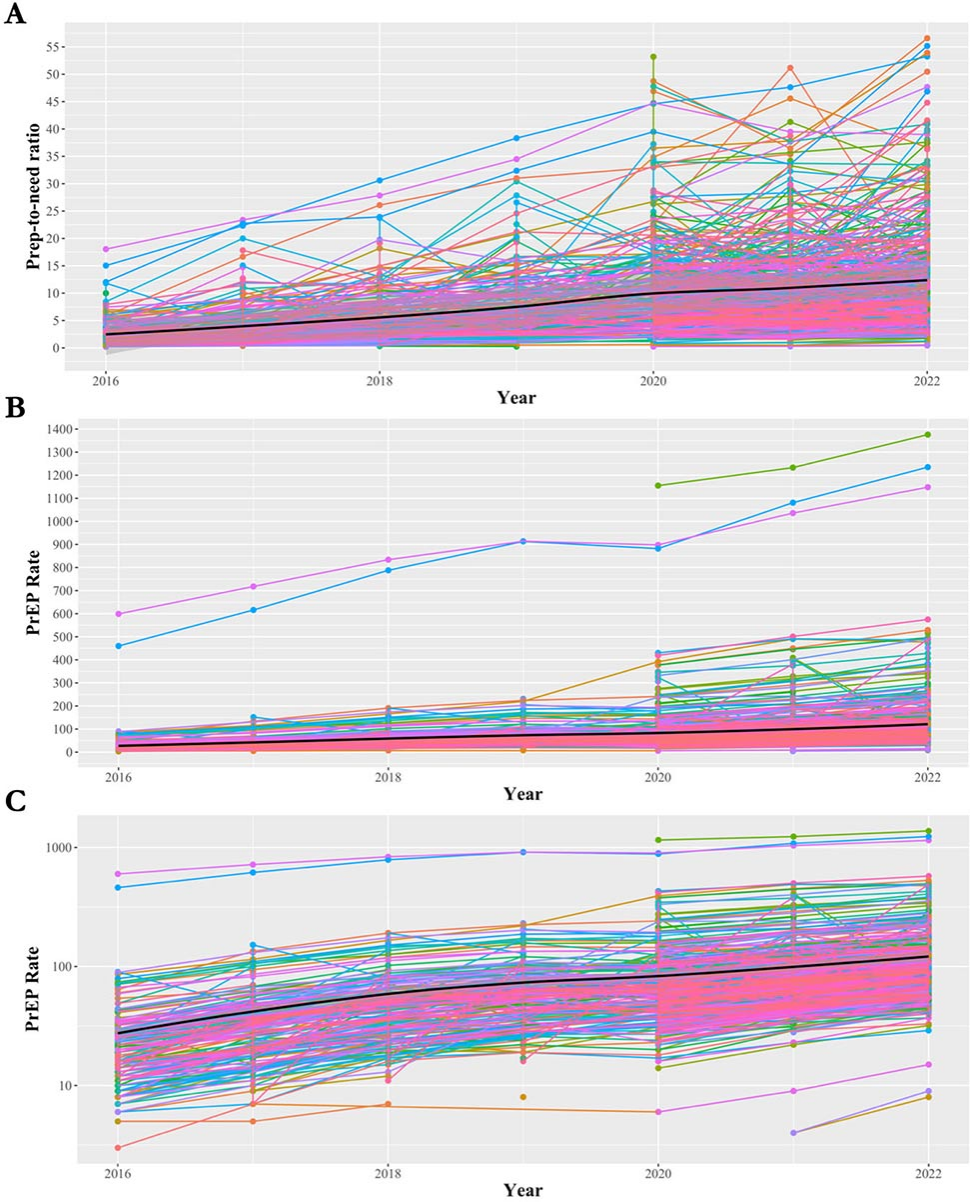
County-level trends in metropolitan PrEP use rate (per 100,000 people) and PrEP-to-need ratio from 2016 through 2022. Panel A. PrEP use rate (per 100,000 people) on linear scale; Panel B. PrEP-to-need ratio on linear scale. Panel C. PrEP use rate (per 100,000 people) on log scale. Note: Variation in y-axis scale by panel.

**Figure 2.**
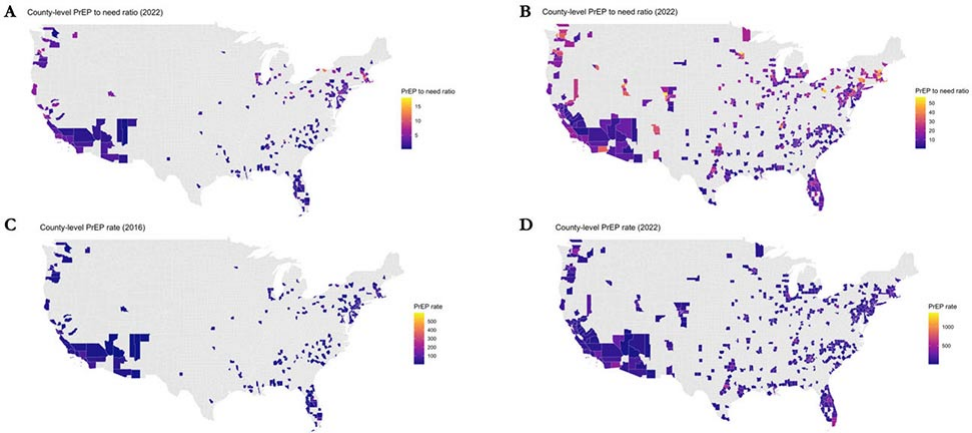
Geospatial trends in county-level PrEP-to-need ratio and PrEP use rates (per 100,000 people) across the metropolitan United States. Panel A. PrEP use rate (per 100,000 people) in 2016; Panel B. PrEP use rate (per 100,000 people) in 2022. Panel C. PrEP-to-need ratio in 2016; Panel D. PrEP-to-need ratio in 2022. Note: Variation in color scale by panel.

**Methods:**

Annual, county-level data for 2016-2022 PrEP-to-need ratios (PNRs) and PrEP rates (recipients per 100,000 people) were from AIDSVu. We included metropolitan counties ( > 100,000 people). We calculated descriptive statistics and temporal trends, performed spatial autocorrelation analyses using global and local Moran’s I, and conducted a difference-in-difference analysis between EHE and non-EHE counties.

**Figure 3.**
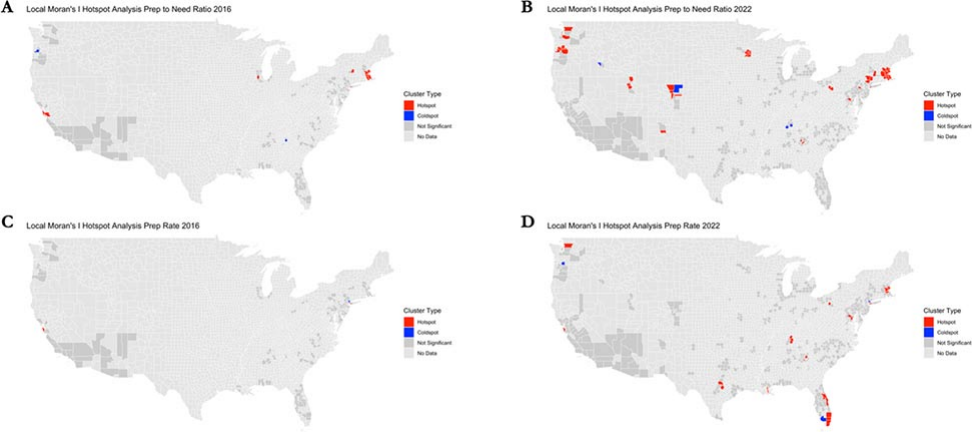
Geospatial analysis of hotspots and coldspots in county-level PrEP-to-need ratio and PrEP rates (per 100,000 people) across the metropolitan United States.

A Local Moran’s I analysis was performed to estimate hotspots (counties of significantly greater PrEP-to-need ratio or PrEP rate compared to the surrounding counties) and coldspots (counties of significantly lower PrEP-to-need ratio or PrEP rate compared to the surrounding counties). Panel A. PrEP-to-need ratio hotspots and coldspots in 2016; Panel B. PrEP-to-need ratio hotspots and coldspots in 2022. Panel C. PrEP use rate hotspots and coldspots in 2016; Panel D. PrEP use rate hotspots and coldspots in 2022.

**Figure 4.**
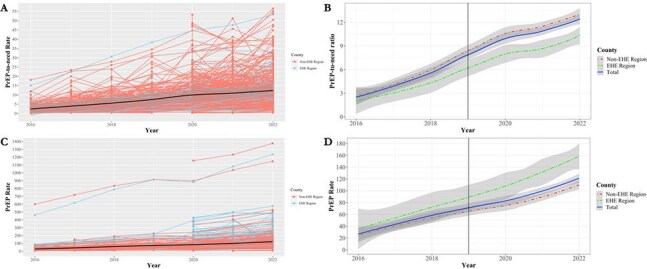
Trends in the PrEP-to-need ratio and PrEP use rate (per 100,000 people) across metropolitan United States counties from 2016 to 2022 by inclusion in the Ending the HIV Epidemic program. Panel A. County-level trends of PrEP use rate (per 100,000 people) stratified by inclusion in the Ending the HIV Epidemic program; Panel B. Averaged county-level trends of PrEP use rate (per 100,000 people) by inclusion in the Ending the HIV Epidemic program. Panel C. County-level trends of PrEP-to-need ratio stratified by inclusion in the Ending the HIV Epidemic program; Panel D. Averaged county-level trends of PrEP-to-need ratio by inclusion in the Ending the HIV Epidemic program. Note: Variation in y-axis scale by panel by outcome.

**Results:**

The average county PNR was 2.5 (range: 0.2-18.0) in 2016 (N=218) and 12.5 (range: 0.4-56.6) in 2022 (N=534); the average PrEP rate was 27.6 (range: 3-599) in 2016 and 121.2 (range: 8-1376) in 2022.

Global Moran’s I analyses of PrEP rates and PNRs for counties with geospatial data were significant in 2016 (N=153) and 2022 (N=455), indicating spatial autocorrelation. Local Moran’s I analyses of PNRs identified 11 hotspots in 2016 and 46 in 2022; PrEP rate analyses found 3 hotspot counties in 2016 and 30 in 2022.

The average PNR in 2016 was 2.1 in EHE counties (N=46) and 2.6 in non-EHE counties (N=172); in 2022, it was 10.3 in EHE counties (N=124) and 13.1 in non-EHE counties (N=410). The average PrEP rate in 2016 was 36.1 in EHE counties and 25.3 in non-EHE counties; in 2022, it was 158.4 in EHE counties and 109.9 in non-EHE counties. The annual PrEP rate of change was 12.3 recipients greater (p=0.04) in EHE counties compared to the baseline annual change; no significant difference was found in the annual PNR change between EHE and baseline trends.

**Conclusion:**

Despite overall increases in PrEP access, marked sub-national disparities persist. While EHE counties made measurable progress, they continued to lag behind non-EHE counties in terms of PNRs. Emerging hotspots, including in the Northwest and the Southeast, demonstrate potential models for expanding access. These findings underscore the need for targeted, local strategies to improve PrEP equity and support national HIV prevention goals.

**Disclosures:**

All Authors: No reported disclosures

